# Formulation of Suppositories of Alum Produced from Bauxite Waste in Ghana for the Treatment of Hemorrhoid

**DOI:** 10.1155/2021/6667562

**Published:** 2021-05-12

**Authors:** Daniel A. Bartels, Raphael Johnson, Marcel T. Bayor, George K. Ainooson, Paul P. S. Ossei, Rashid K. Etuaful, Richard Buamah

**Affiliations:** ^1^Department of Pharmaceutics, College of Health Sciences, Kwame Nkrumah University of Science and Technology, Kumasi, Ghana; ^2^Department of Pharmacology, College of Health Sciences, Kwame Nkrumah University of Science and Technology, Kumasi, Ghana; ^3^Department of Pathology, College of Health Sciences, Kwame Nkrumah University of Science and Technology, Kumasi, Ghana; ^4^Department of Civil Engineering, College of Engineering, Kwame Nkrumah University of Science and Technology, Kumasi, Ghana

## Abstract

The study sought to formulate and evaluate suppositories using a locally produced brand of alum (Aw) obtained from bauxite waste generated at Awaso bauxite mine in the Western-North region of Ghana, for use in the treatment of hemorrhoids. The suppositories were formulated using shea butter modified, respectively, with amounts of beeswax and theobroma oil. In another development, theobroma oil was modified with different concentrations of beeswax. Drug-base interactions were investigated using attenuated total reflection-Fourier transform infrared (ATR-FTIR) spectroscopy. The suppositories were prepared using the hot melt and trituration methods. Quality control checks were carried out on the formulations. The evaluated parameters included physical characteristics (texture, presence or absence of entrapped air, and contraction holes), weight uniformity, disintegration time, drug content, and *in vitro* release profile of the alum from the formulated suppositories. An *in vivo* analysis was carried out on the most suitable formulation to ascertain its efficacy on inflamed tissues using croton oil-induced rectal inflammation in a rat model. A critical examination of the ATR-FTIR spectra revealed no drug-base interactions. The suppository formulations passed all Pharmacopoeia stated tests. The *in vivo* study revealed the use of suppositories ameliorated the croton oil-induced hemorrhoid in the rectoanal region of the rats.

## 1. Introduction

Hemorrhoids, also called piles, are swollen veins in the anus and lower rectum, similar to varicose veins. Hemorrhoids can develop inside the rectum (internal hemorrhoids) or under the skin around the anus (external hemorrhoids). It occurs frequently as an inflammatory process of the hemorrhoidal plexus [1]. They are often not associated with any symptoms and people may not know they have them. When symptoms do occur, they present as bleeding and/or pain on passing stool, sense of incomplete bowel emptying, a lump around or inside the anus, itchiness or soreness around the anus, and mucus discharge from the anus [2]. Hemorrhoids affect about 4.4% of the general population worldwide, irrespective of gender. However, it is more frequent in females than in males with age between 45 and 65 years. Caucasians are affected more frequently than African Americans, and higher socioeconomic status is associated with increased prevalence [3]. Long-term constipation, diarrhoea, long duration of sitting, strenuous manual performance involving lifting, aging, obesity, overuse of laxatives, and pregnancy are considered some of the risk factors that could lead to the development of hemorrhoids [2, 4]. Despite its low morbidity, hemorrhoids have a high impact on quality of life.

Management of symptomatic hemorrhoids ranges from nonoperative medical interventions (drugs, dietary, and lifestyle modification) and office-based procedures (rubber band ligation, sclerotherapy, and infrared coagulation) to surgery (hemorrhoidectomy, stapled hemorrhoidopexy, and Doppler-guided haemorrhoid artery ligation) [2]. Lifestyle modification includes increasing oral fluid intake, reducing fat consumption, avoiding straining, and regular exercise. Increasing the intake of fibre-rich diet is recommended [5]. Pharmacological treatment of haemorrhoid aims at reducing inflammation, pain, and bleeding. Topical dosage forms such as creams, ointments, and suppositories containing steroids (corticosteroids) or nonsteroidal anti-inflammatory agents (NSAIDS: ibuprofen, aspirin, and diclofenac), either alone or in combination with antibiotics or anaesthetics (such as lidocaine, benzocaine, and dibucaine), have been shown to be effective. However, their prolonged use is limited due to the high incidence of side effects [6–8]. Creams and ointments are commonly applied to external hemorrhoids for temporary relief. Rectal suppositories are better for internal hemorrhoids. Suppositories are usually inserted after a bowel movement, so the effect can last longer because they break down more slowly, releasing medication over a longer period of time. The medicine is absorbed by the rectal tissue and can help all discomfort and pain caused by hemorrhoids. Suppositories are prepared using bases as vehicles. The bases are of two types: oleagineous/fatty bases (theobroma oil or cocoa butter and hydrogenated vegetable oils) and water miscible or soluble (glycerinated gelatin and polyethylene glycol polymers also known as macrogols). The type of base used depends on the physicochemical properties of the drug to be incorporated [9, 10]. Commercially available fatty bases which are considered as cocoa butter substitutes include Dehydag®, Hydrokote®, Suppocire®, and Witepsol®. Hydrocortisone and NSAID-containing suppositories such as Anusol-HC®, Anucort-HC®, Voltarol®, and Voltarene® are formulated using hydrogenated vegetable oils [9, 11]. These bases are very stable to oxidation, have a longer shelf-life, set quickly, and do not exhibit polymorphism. They are, however, costly and have to be imported for use.

Alum is a double salt of sulphate bonded to potassium and aluminium coordinated to molecules of water [KAl(SO_4_)_2_·12·H_2_O]. Alum can be obtained from minerals such as kalinite, alunite, leucite, and bauxite [12]. It is mostly known for its use in the purification of water [13]. However, alum has other uses such as making paper, fireproof textiles, and deodorants [14]. In recent years, scientific studies have proven that alum has pharmacological properties such as antihemorrhagic, antimicrobial, and anti-inflammatory [15]. Nonsteroidal anti-inflammatory drugs are more tolerable to most people than corticosteroids. The anti-inflammatory and antihemorrhagic effects could make alum a useful active ingredient in the treatment of hemorrhoids. Alum is commercially produced by a hydrometallurgical process. This process begins with extraction of alumina (solid Al_2_O_3_) from bauxite waste with the help of an aqueous solution of sulphuric acid. The resultant solution is then reacted with potassium sulphate to produce potassium aluminium sulphate [16]. In Ghana, bauxite is essentially mined at Awaso (Sefwi), a town in the Western-North of Ghana. This mine has been in operation for over sixty years, and plans are far ahead to increase the production of bauxite in the coming years. This development may result in the plant generating more waste, expected to be over 100,000 tons per annum with a potential risk of inundating the mine site [17]. Therefore, Etuaful and Buamah developed an improved protocol for producing alum from the Awaso bauxite waste. The protocol has been found to be environmentally friendly, cheaper, and less burdensome and produces a high yield of alum (Aw) [18]. Potassium alum is considered by the United States Food and Drugs Authority as a generally recognized as a safe (GRAS) substance and has been used in food and cosmetics for human use [19].

This work sought to evaluate the pharmaceutical application of this locally produced alum in a suppository for the treatment of hemorrhoids. Since alum has been used in large amounts for the purification of water for human consumption with no noticeable side effects, its use for haemorrhoid treatment will not cause undesirable side effects associated with corticosteroids and NSAIDs. They can also be used for a prolonged period due to the low amount that will be required for treatment. Alum is highly hydrophilic and therefore requires a fatty base for its formulation. The suppositories were formulated using theobroma oil, shea butter, and beeswax. Shea butter is locally abundant and cheap with a low melting point. As such, beeswax was employed as a hardening agent. Theobroma oil is known to be a standard fatty base for suppositories, but it is costly. Therefore, combinations of these three available bases were evaluated as bases for the formulation of suppositories with locally produced alum. The suppositories were evaluated for quality according to standard specifications and tested for their antihemorrhoidal effect in rat models.

## 2. Materials and Methods

### 2.1. Materials

The local alum (Aw) was obtained from the Civil Engineering Department of the College of Engineering, KNUST, Kumasi. White beeswax was obtained from the Department of Pharmaceutics, KNUST, Kumasi. Theobroma oil (cocoa butter) was purchased from Tree of Life, Ghana Limited, Accra. Diclofenac sodium powder was obtained as a gift from Danadams Pharmaceutical Industry, Ghana. Shea butter was purchased from Savelugu in the northern part of Ghana. All other chemical reagents were obtained from the Department of Pharmaceutics, KNUST, Kumasi.

### 2.2. Methods

#### 2.2.1. Identification and Authentication of the Alum Sample

The physical characteristics of the alum were determined by the appearance, colour, smell, feel, pH, and solubility. The presence of major elements (Al^3+^, K^+^, and SO_4_^2−^) was investigated by qualitative chemical tests.

*(1) Identification Test of Aluminium Ion (Al* ^*3+*^). One gram of the powdered alum (Aw) was dissolved in 10 ml of distilled water in a test tube. 0.1 M of sodium hydroxide solution (NaOH) was added dropwise to 1 ml of the aqueous solution containing the alum. About 1 ml of aqueous ammonia (NH_3_) solution was added to the aqueous solution. The presence of a white precipitate indicated the presence of aluminium [20]. The procedure was repeated two more times.

*(2) Identification Test of Potassium Ion (K* ^*+*^). A cotton swab was moistened at one end with distilled water. The moistened end of the cotton swab was then dipped into a container containing the powdered alum (Aw) and then introduced to a nonluminous flame from a Bunsen burner. A violet colour change of the flame indicated the presence of potassium ions present in the alum [20]. The experiment was done in triplicate.

*(3) Identification Test of Sulphate Ion (SO*_*4*_^*2−*^). About 1 ml of dilute hydrochloric acid was added to 2 ml of the aqueous solution of the alum. 0.2 M barium chloride (BaCl_2_) solution was then added to the resulting solution dropwise. The formation of a white precipitate indicated the presence of sulphate ion [20]. The experiment was repeated two more times.

*(4) Determination of pH of Alum Solution*. One gram of the grounded powder of alum (Aw) was weighed and dissolved in 10 ml of distilled water. It was then filtered and the pH of the filtrate was determined using a previously calibrated Eutech® pH meter (pH 510, pH/mV/°C meter, Singapore) under room temperature [20]. This test was done in triplicate.

*(5) Determination of Solubility of Alum in Water*. The solubility of the powdered alum (Aw) in water was determined by weighing accurately 65 g into 100 ml of distilled water at 25 C ± 2°C. It was well shaken at time intervals for the first 30 minutes. They were then kept uninterrupted for 2 hours at room temperature. The mixture was filtered and 7.3 ml of the filtrate was diluted to 300 ml. The diluted solution was then analyzed by complexometric back titration with ethylenediaminetetraacetic acid (EDTA). In this method, 10 ml of the diluted filtrate was pipetted into a conical flask. 50 ml of 0.01 M EDTA was added and boiled for about 5 minutes. The mixture was left to cool to room temperature. 5 drops of solo chrome black were added to the mixture, and a blue colour change was observed. About 1 ml of 10% ammonia solution was added to the mixture. The resultant solution was then titrated with 0.01 M zinc sulphate solution until a pink colour was observed as an endpoint, and the titre value was recorded. The difference between the EDTA volume added and the titre value was then calculated (EDTA volume (ml) – titre value of zinc sulphate (ml)). The amount of alum was then calculated using the following formula:fd1

(1) $$\begin{array}{c} \text{mass of alum}=23.73\text{ mg}\times \left(\text{EDTA vol}\left(\text{ml}\right)-\text{titre value of zinc sulphate}\right)\times \text{dilution factor}\left(\text{DF}\right). \end{array}$$

This experiment was carried out in triplicate [21].

The solubility of the powder of the alum (Aw) in hot water was also determined by weighing accurately 65 g into 100 ml of distilled water at 50°C ± 2°C. The procedure as stated above was followed.

*(6) Identification of Alum, Shea Butter, Theobroma Oil, and Beeswax by Infrared Spectroscopy*. Attenuated total reflection-Fourier transform infrared (ATR-FTIR) spectroscopy (ATR-FTIR spectrometer, 200-X, SN: 200043, USA) was used to identify the presence of major peaks in the alum, shea butter, theobroma oil, and beeswax in the wavenumber range of 400 cm^−1^ to 4000 cm^−1^ and compared with standards. The diamond crystal sensor of the ATR-FTIR was cleaned with isopropanol to get rid of every impurity present on the sensor that will affect the result. With the help of a spatula, a little amount of alum powder was placed on the diamond crystal and the spectrum was generated in the stated wavenumber range. The same was done for shea butter, theobroma oil, and beeswax.

#### 2.2.2. Drug-Base Interactions

Attenuated total reflection-Fourier transform infrared (ATR-FTIR) spectroscopy method was used to investigate any drug-base interactions. A physical mixture of alum (Aw) and shea butter was prepared in a 1 : 1 ratio and placed on the diamond crystal and scanned for the spectrum in the wavenumber range of 400 cm^−1^ to 4000 cm^−1^. This was repeated for theobroma oil mixed with alum, and beeswax was mixed with alum. Again, shea butter was mixed with theobroma oil and subsequently mixed with alum in a ratio of 1 : 1 : 1 and the spectrum was obtained. This was repeated for shea butter with beeswax and alum. The ATR-FTIR spectra were analyzed for possible drug-base interactions by observing missing peaks, elongation, and shortening of peaks.

#### 2.2.3. Determination of the Displacement Value of the Alum

Six suppositories of each base (shea butter with beeswax, theobroma oil with beeswax, or shea butter with theobroma oil) were prepared and weighed (a mg) using a 2 g mold (method explained in detail under Section 2.2.4). These are referred to as blank suppositories. Six medicated suppositories containing 40% of the alum were also prepared for the above-stated bases and weighed (b mg). The amount of the bases (c mg) and alum (d mg) in the medicated suppository was determined [22]. The displacement value of the alum in a particular base was calculated using the following formula:

(2) $$\begin{array}{c} \text{displacement value}=\frac{\text{d}\left(\text{mg}\right)}{\text{a}-\text{c}\left(\text{mg}\right)}. \end{array}$$

#### 2.2.4. Preparation of Suppositories

Forty suppositories of each of the bases and modifications (Table 1) were prepared using the hot melt and trituration methods. Ten formulations were investigated by (1) modifying shea butter with different amounts of beeswax; (2) modifying shea butter with different amounts of theobroma oil, and (3) modifying theobroma oil with varying amounts of beeswax.

Blank suppository using, for example, shea butter and beeswax was prepared using the 2 g mold and weighed. The displacement value stated in Table 2 for shea butter and beeswax was used to calculate the total amount of base required for the formulation of the forty suppositories by using the following equation [23]:

(3) $$\begin{array}{c} \text{amount of total base}\left(\text{B}\right)=\left(N\times y\right)-\;\left(N\times \frac{D}{\text{DV}}\right), \end{array}$$

where *N* is the number of suppositories to be formulated, *y* is the weight of blank suppository (2.0317 g), *D* is the amount of drug in one suppository (0.5 g), and DV is the displacement value of base (1.73). Of the total base, 5% constituted the beeswax. The constituents of the formulations are as shown in Table 1.

A 2 g suppository mold (D-7166, Erwema Pharmatechnik, Germany) was disassembled and washed clean and dried. The mold was lubricated with the help of cotton wool and soap spirit. The alum (Aw) was weighed using an analytical balance (SN: AE 436647 Adam Equipment, UK). The beeswax plus shea butter base formulations were prepared by first heating the beeswax at about 64°C on a water bath (Sanyo, OMT Oven, Gallenkamp, UK) until it liquefied completely. The shea butter was shredded, weighed, and added to the liquefied beeswax and stirred steadily. Care was taken to prevent overheating the shea butter. The procedure was repeated for the beeswax plus theobroma oil base formulations, but with the formulations of the *theobroma* oil and the *theobroma* oil plus shea butter, they were shredded, weighed, and heated together in a stainless steel container on a water bath at a temperature of 37°C until a uniform mixture was obtained. The alum was incorporated into a little of the base on a warm tile and then stirred with the rest of the base until a homogeneous mixture was obtained. The molten mass was left to set and poured into the mold until overfill. The mold was then refrigerated for about 30 minutes for solidification. The excess base was scraped from the top of the mold with a warm spatula. The mold was disassembled and the suppositories were removed, packed, and stored until further use.

### 2.3. Quality Evaluation of Formulated Suppositories

The suppositories were evaluated in accordance with the requirements of the British Pharmacopoeia [24].

#### 2.3.1. Physical Characteristics of Suppositories

*(1) Sensory Evaluation*. The formulated suppositories were assessed for colour and surface parameters such as texture, appearance, feel, and shape to ensure product-to-product consistency. The odour of the prepared suppositories was verified by smelling each of the various formulations and recorded.

*(2) Surface Condition*. The surfaces of the suppositories were examined for brilliance, dullness, mottling, cracks, dark regions, axial cavities, bursts, air bubbles, and holes, and observations were noted.

#### 2.3.2. Uniformity of Weight Test on Suppositories

Twenty suppositories from each formulation were randomly selected and collectively weighed. They were also weighed individually. The mean and individual weight of the suppositories were determined as A and B, respectively. The individual suppository weight was deducted from the mean weight of the suppositories (A-B). The percentage deviation of each suppository from the mean was also calculated using the following formula:

(4) $$\begin{array}{c} \frac{\left|\text{A}-\text{B}\right|}{\text{A}}\times 100\%, \end{array}$$

where A is the mean weight of the suppositories and B is the individual weight.

For all weights of suppositories, not more than two suppositories must deviate from the mean weight by more than 5% and none of the suppositories must deviate by more than 10% [25].

#### 2.3.3. Disintegration Tests of Suppositories

The USP tablet disintegration apparatus (Erweka Type ZT 3/1, GmbH, Heusenstamm, Nr 68318, Germany) was used for the test. Six suppositories from each batch were placed in the basket rack of the disintegration apparatus and a plastic disk was placed on each. The time it took for all six suppositories to melt or soften at 37°C ± 2°C in 1000 ml of distilled water was recorded. The mean of six determinations and the standard deviation were calculated.

#### 2.3.4. Content Uniformity of Suppositories

The drug content was determined according to the method used by Baria et al. [26] with few modifications. Ten individual suppositories were randomly selected from the formulations (Table 1) and assayed individually for their drug contents. For the assay, the suppositories were weighed individually and their weights were recorded. Each suppository was put in a 100 ml beaker and was dispersed in deionized water (50 ml) on a water bath. The resultant mixture was made up to 100 ml mark with more deionized water and kept at 50°C. The solution was filtered with a Whatman filter paper with the aid of a funnel into a 200 ml conical flask. The filtrate was analyzed using the complexometric back titration method as described under the solubility test. This was repeated for the ten randomly selected suppositories. The average content and standard deviation were determined.

#### 2.3.5. Dissolution Test on Suppositories

The dissolution test was carried out using the USP apparatus II (Erweka Type DT6, GmbH, Heusenstamm, Nr 68045, Germany). The experiment was performed using 900 ml deionized water of pH 6.8, a paddle speed of 50 revolutions per minute, and a temperature of 37°C ± 0.5°C. At a specified time interval, 10 ml of the dissolution medium was pipetted from the vessel and filtered immediately into a test tube. 10 ml of fresh dissolution medium was withdrawn from the reservoir to replace the 10 ml withdrawn from the dissolution vessel. The filtrate was then diluted with distilled water to the 100 ml mark of the volumetric flask and analyzed by complexometric titration as described above under the solubility/content test. The concentration of the alum released was calculated. The percentage release and cumulative percentage release were then determined. The procedure was repeated at specific time intervals of 15, 30, 45, 60, 90, 120, 150, and 180 minutes. A plot of cumulative percentage drug release against time was established.

### 2.4. Experimental Animals

Twenty-five (25) male Sprague Dawley rats (5 to 6 months old) were purchased from the Animal Unit of Noguchi Memorial Institute for Medical Research, Accra, and kept in stainless steel cages of dimensions 34 cm × 57 cm × 40 cm at a population of 5 animals per cage, in the animal house of the Department of Pharmacology, Faculty of Pharmacy and Pharmaceutical Sciences, KNUST, Kumasi, to acclimatize for 7 days. Commercial feed (Agricare, Kumasi) and water were provided *ad libitum* throughout the experiment. Room temperature of 29°C was maintained within the cages and a 12-hour light-dark cycle was maintained using overhead incandescent illumination [27]. All experiments were conducted in accordance with protocols from the National Research Council (US) Committee for the Update of the “Guide for the Care and Use of Laboratory Animals”-8th edition (2011). All protocols used were approved by the Ethics Committee of the Department of Pharmacology, Kwame Nkrumah University of Science & Technology, Kumasi, Ghana.

### 2.5. Evaluation of the Antihemorrhoid Property of the Alum Suppository

Evaluation of antihemorrhoidal properties was carried out as described by Azeemuddin et al. [28] with some modifications. To do this, Sprague Dawley rats (220–240 g) were randomly selected and put into five groups of five (G1 to G5, *n* = 5). Group G1 was the naïve (untreated) group. Groups G2, G3, G4, and G5 were pretreated with sterile cotton swabs (4 mm diameter) soaked in 100 *μ*L of croton oil inserted into the anus (rectoanal portion, 20 mm from anal opening) and kept for 10 seconds. The cotton swabs were removed and thereafter groups G3, G4, and G5 received daily for 5 days, bland (0.5 g = vehicle control), diclofenac (15 mg = positive control), and alum (100 mg = ST3) suppositories, respectively. The rats were weighed and sacrificed on the fifth day.

Rectoanal tissue (20 mm in length from the anal opening) of each rat was taken off and weighed. The severity of the hemorrhoids was determined using the rectoanal coefficient (RAC) calculated using the following formula:

(5) $$\begin{array}{c} \text{rectoanal coefficient}\left(\text{RAC}\right)=\frac{\text{weight of rectoanal tissue}\left(\text{mg}\right)}{\text{body weight}\left(\text{g}\right)}. \end{array}$$

Rectoanal coefficient (RAC) can be defined as the ratio of the rectoanal part of the test animal in milligrams (mg) to the body weight of the same animal in grams (g). The smaller the RAC value, the more efficacious the remedy.

Histopathological analysis was carried out on the excised tissues, to study the disease state at the microscopic level. The tissues were fixed in 10% neutral buffered formalin solution for examination using a light microscope (Olympus BX 51TF, Olympus Corporation, Tokyo, Japan).

### 2.6. Statistical Analysis

Results on the effect of alum (Aw) suppositories on croton oil-induced hemorrhoids were expressed as the means ± standard error of mean (SEM). Statistical significance of the differences between the means was determined by one-way analysis of variance (ANOVA) using Kruskal–Wallis test, followed by Dunn's multiple comparison test, using GraphPad Prism (GraphPad Prism, version 8 for Windows, San Diego, CA, USA). A *P* < 0.05 was considered statistically significant.

## 3. Results and Discussion

Suppositories consist of a base or a combination of bases (vehicle) and the active pharmaceutical ingredient (drug). The choice of a base depends on the partition coefficient of the drug in the base and the biological fluid. When a drug has a high vehicle-to-water partition coefficient, the tendency to leave the vehicle is minimal, and thus, the release rate into the rectal fluid is low. This is not favourable for rapid absorption. On the other hand, certain lipid solubility is required for penetration through the rectal membrane. The optimal balance between these two requirements should always be established. Thus, as a general rule, a drug with low fat solubility and high water solubility requires a fatty base as a vehicle for its formulation into a suppository [9, 10]. Alum is highly water-soluble; hence, a fatty base was needed for its formulation. Cocoa butter (theobroma oil) and shea butter are fatty bases. Cocoa butter is costly while shea butter is cheap and readily available locally but has a low melting point. Thus, to improve upon its melting properties, beeswax was added as a hardening agent.

### 3.1. Identification and Authentication of the Alum Sample

**Figure 1:**
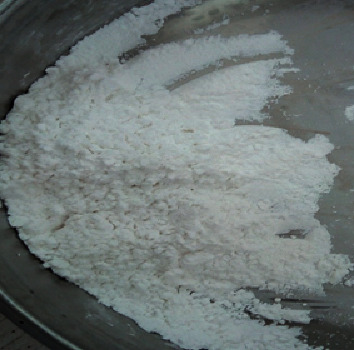
Powdered alum sample produced from bauxite waste in Ghana.

The physical test results revealed that the alum was white in colour and odourless with a smooth and glassy surface texture. It was very hard to be fractured (Figure 1 and Table 2).

The pH evaluation revealed the alum was acidic. All these physical properties complied with the identity of alum by the British Pharmacopoeia [20]. Identification test is vital in determining the authenticity of any substance. Thus, in order to determine how authentic the alum was, various identification tests as stated in the British Pharmacopoeia were carried out. The alum reacted with sodium hydroxide to produce a white precipitate [20]. This indicated the presence of aluminium ions. The resultant solution reacted with ammonia solution to produce a gelatinous precipitate confirming the presence of aluminium in alum. The addition of dilute hydrochloric acid to alum produces white precipitates to indicate the presence of sulphate ion. Upon addition of 2 M barium chloride to the resulting solution, the white precipitate was maintained confirming the presence of the sulphate ions. Introducing alum to a nonluminous flame to produce a pale violet flame indicates the presence of potassium.

In the current research, the alum sample produced a similar outcome as stated in the British Pharmacopoeia [20] for the identification of alum as well as that observed by Birnin-Yauri and Musa [29].

Solubility is a preformulation parameter that has to be assessed for all drugs. It influences the dissolution characteristics of the drug in a dosage form and hence its bioavailability. Solubility has also been used as one of the criteria for the classification of drugs according to the Biopharmaceutics Classification System (BCS) [30]. The solubility test results showed that 13.67 ± 0.01 g and 30.80 ± 0.01 g of alum completely dissolved in 100 ml of distilled water at 25°C and 50°C, respectively. These values suggest that the sample is freely soluble in water and its solubility increases with increasing temperature. The values were, however, slightly lower than those stated in the literature of 14 g/100 ml (20°C) and 36.80 g/100 ml (50°C) [31]. This difference in solubility may be due to the source of the alum produced.

### 3.2. Displacement Value of the Alum

Traditionally, suppositories are prepared using suppository molds which can generally hold 1 gram or 2 grams of base. That is, the volume of the mold generally remains constant and cannot change. Whenever we add drug or other excipients to the base, the drug occupies some of the space and displaces a certain quantity of base. The displacement value is therefore an indicator of the amount of the base that is displaced to accommodate the medicament to be incorporated. The displacement value of a drug must be determined in a particular base in which it is incorporated. It is essential in ensuring that the correct amount of drug is contained within a defined quantity of a dosage form. The displacement value and the amount of drug to be incorporated enable one to determine the amount of base required for the formulation. The displacement values of alum (Aw) in all suppository formulations were evaluated and the results are given in Table 3. The displacement values were not significantly different for the different bases (*P* > 0.05).

### 3.3. Investigation of Possible Drug-Base Interactions Using FT-IR Spectroscopy

**Figure 2:**
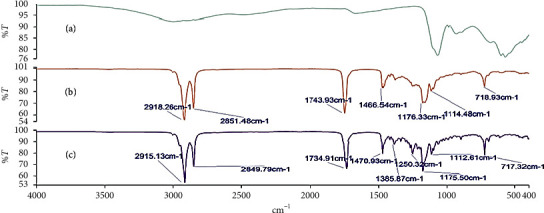
FT-IR spectra of (a) alum, (b) theobroma oil, and (c) theobroma oil + alum (1 : 1).

**Figure 3:**
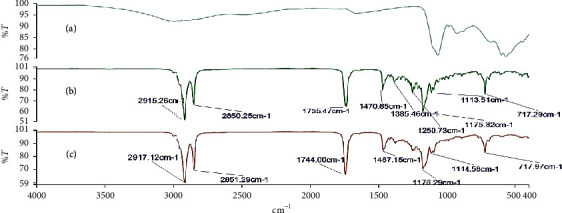
FT-IR spectra of (a) alum, (b) shea butter, and (c) shea butter + alum (1 : 1).

**Figure 4:**
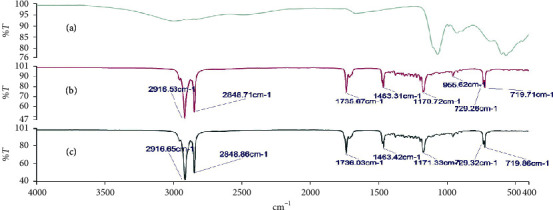
FT-IR spectra of (a) alum, (b) beeswax + alum (1 : 1), and (c) beeswax.

**Figure 5:**
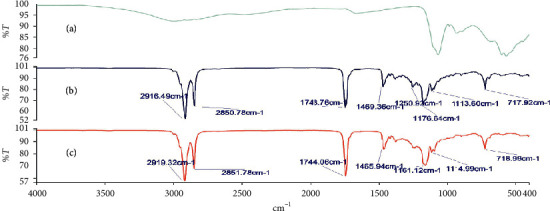
FT-IR spectra of (a) alum, (b) shea butter + theobroma oil + alum (1 : 1 : 1), and (c) shea butter + theobroma oil (1 : 1).

**Figure 6:**
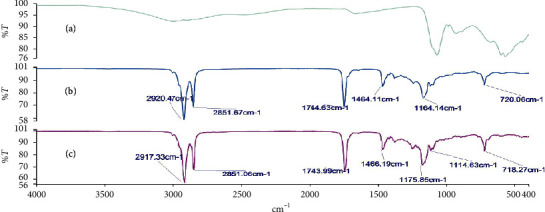
FT-IR spectra of (a) alum, (b) beeswax + shea butter + alum (1 : 1 : 1), and (c) beeswax + shea butter (1 : 1).

Fourier transform infrared (FT-IR) spectroscopy was carried out on the alum powder, the theobroma oil, beeswax, shea butter, and mixtures of these bases to ascertain the compatibility of the drug substance with the different bases. Figures 2–6 show the FT-IR spectra of the alum, theobroma oil, beeswax, shea butter, and different combinations, respectively. In developing a rectal suppository dosage form, it is very essential to carry out drug-base interaction studies to help eliminate the issues of incompatibility and stability and ensure the release of the drug from the base used in the formulations.

Theobroma oil, shea butter, and beeswax gave identical spectra. They showed peaks in the ranges of 2920.47 cm^−1^ to 2915.29 cm^−1^, 2851.78 cm^−1^ to 2848.71 cm^−1^, 1744.63 cm^−1^ to 1735.47 cm^−1^, 1470.65 cm^−1^ to 1464.11 cm^−1^, 1176.33 cm^−1^ to 1161.12 cm^−1^, and 719.71 cm^−1^ to 718.71 cm^−1^ which corresponds to C-H (CH_2_), C-H (CH_3_) stretching vibrations, -C=O stretching vibrations of acids and esters [32], -C-H-(CH_2_CH_3_) bending vibrations of CH_2_ and CH_3_ aliphatic groups [33, 34], and C-O bond of esters and bending vibrations of a methylene group present [32], respectively. Moreover, their respective physical mixtures revealed absorption bands in the ranges of 2917.33 cm^−1^ to 2915.13 cm^−1^, 2861.06 cm^−1^ to 2848.71 cm^−1^, 1744.00 cm^−1^ to 1734.91 cm^−1^, 1470.93 cm^−1^ to 1463.31 cm^−1^, 1175.65 cm^−1^ to 1170.72 cm^−1^, 114.63 cm^−1^ to 114.48 cm^−1^, and 719.71 cm^−1^ to 717.27 cm^−1^. Comparative analysis shows that there are no significant differences (*P* > 0.05) in the absorption bands, indicating no chemical interaction between the bases and the drug. There were no major shifts, elongations, broadening, widening, and shortening of the prominent peaks of the bases. Furthermore, there were no new peaks found in the physical mixtures of drug and the bases. Comparatively, the interaction was independent of the type of base or mixture of bases.

### 3.4. Physical Appearance of the Formulated Suppositories

The suppositories prepared were very smooth and torpedo-shaped and had no trapped air when they were cut open. All the suppositories formulated with shea butter and beeswax had a characteristic smell due to the high amount of shea butter which has a strong characteristic odour and the *theobroma* oil-formulated suppositories had a chocolate smell which is characteristic of the smell of *Theobroma cacao*. The suppositories were satisfactory in appearance according to British Pharmacopoeia specification [20].

### 3.5. Quality Assessment of the Formulated Suppositories

The Pharmacopeia requires weight variation, disintegration, and content uniformity tests to be conducted on suppositories.  Table 4 gives the results of the tests conducted.

The suppositories had uniform weight in accordance with BP specification [24]. The uniform weight predicts a uniform distribution of the alum in the suppositories and that there is no variation in the amount of alum in each suppository. The uniformity of content test assesses suppository-to-suppository and batch-to-batch consistency for production technique optimization. The requirements for drug content are met if the amount of the drug substance in each of the 10 dosage units as determined by the Content Uniformity method lies within the range of 85.0 to 115.0% of the label claim. All formulated suppositories were found to be within acceptable limits [24]. This confirms the uniformity of the dose and hence an anticipated uniform therapeutic response.

According to the British Pharmacopoeia [24], disintegration of suppositories occurs when the test sample softens or dissolves completely. For fatty base suppositories, this is expected to occur within 30 minutes. All components, except for alum (shea butter, theobroma oil, and beeswax) are oleaginous in nature and soften on exposure to temperature. All the formulated suppositories passed the disintegration test. However, the disintegration time for the shea butter and beeswax combination increased with increasing amounts of beeswax, which is as expected due to the hardening effect of the beeswax. This trend was also observed by Oladimeji Francis and Adeyemi [35] when they formulated metronidazole suppositories using shea butter and cocoa butter modified with 20% w/w of beeswax. Saleem et al. [36] also observed that the disintegration time increased when tramadol hydrochloride suppositories were prepared with cocoa butter modified with 1% w/w and 3% w/w beeswax.

### 3.6. *In Vitro* Drug Release of the Suppositories

**Figure 7:**
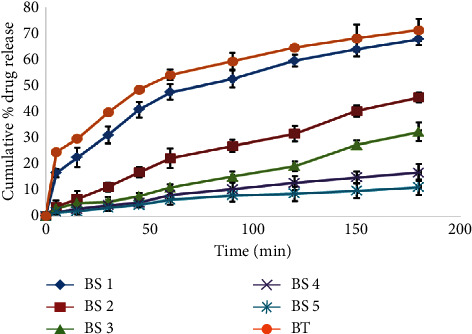
A plot of cumulative percentage alum release with time for suppository formulations BS1, BS2, BS3, BS4, BS5, and BT. (BS: beeswax + shea butter; BT: theobroma + beeswax). Error bars represent the standard deviation of six replicates.

For suppositories, the dissolution of medicaments in the rectal fluid precedes absorption and this can be tested *in vitro*. Dissolution of drugs is the rate-determining step in the absorption of drugs and subsequent pharmacological activity. According to the British Pharmacopoeia [24], the drug release from nonmodified dosage forms at time 45 minutes in the dissolution medium should not be less than 70%. From the result as shown in Figure 7, the suppository formulations BS1, BS2, BS3, BS4, BS5, and BT had the unsatisfactory release of the alum (Aw) into the solution at time 45 minutes. One of the first requisites for a suppository base is that it should remain solid at room temperature but soften, melt, or dissolve readily at body temperature so that the drug is fully available soon after insertion [9]. The low release could be due to the incorporation of beeswax to improve upon the melting properties and mechanical strength of the shea butter and theobroma oil.

Beeswax is highly hydrophobic; therefore, incorporating it into the formulation increases the hydrophobicity of the whole formulation. The increase in the hydrophobic nature decreased the softening tendency of the bases in the medium (water) and hence affected the release of the drug by trapping the alum in itself [37]. The presence of beeswax caused a dispersion of the suppositories in the dissolution medium and thus impacted negatively on the release rate. Even though the release of alum from the suppository was low at 45 minutes, the release could reach 71.14 ± 4.30% in 180 minutes for the beeswax-theobroma oil. This indicated a controlled or slow release property of the formulations and a high probability for their use as controlled release suppositories. Saleem et al. [36] and Oladimeji Francis and Adeyemi [35] observed similar slow release of tramadol hydrochloride and metronidazole, respectively, from cocoa butter and shea butter modified with different amounts of beeswax. Saleem et al. [36] attributed the slow release of tramadol hydrochloride to high lipophilicity of the base, high water solubility of tramadol hydrochloride, nonmiscibility of the base with the dissolution media, and absence of additives or surface-active agents. The alum is also highly soluble in water and was incorporated in a lipophilic base without any additive and surface-active agent, hence sharing the same fate of slow release. The alum release was, however, better than that of tramadol hydrochloride. The slow release of the alum from the base was correlated with the amount of beeswax added.

**Figure 8:**
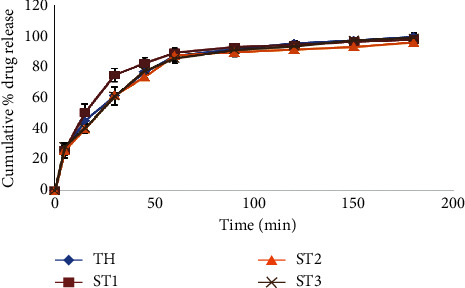
Plot of cumulative percentage release of alum against time for suppository formulations TH, ST1, ST2, and ST3. (ST: shea butter + theobroma oil; TH: theobroma oil). Error bars represent the standard deviation of six replicates.

The release of the alum from the suppository formulations TH, ST1, ST2, and ST3 at time 45 minutes was 76.71 ± 3.52%, 82.43 ± 3.3%, 73.53 ± 2.34%, and 77.75 ± 2.92%, respectively (Figure 8).

The shea butter and cocoa butter suppositories are lipophilic suppositories that melt at rectal temperature to release medicament. Due to this, their release is greatly affected by temperature as their softening time shortens. The alum “Aw” is a freely water-soluble drug and will have a high tendency to migrate from a lipophilic base into an aqueous dissolution medium. The results showed that the suppository formulations without beeswax (TH, ST1, ST2, and ST3) could be considered as an immediate release suppository dosage form [24]. The amount of alum released at 45 minutes from the shea butter and cocoa butter combinations was higher than other drug release studies using similar bases [35–40]. Some of these studies had the inclusion of different amounts of Tween 80 as a surfactant. Theobroma oil is costly and scarce in our part of the world; hence, this can increase the overall cost of the production of suppositories of alum. Formulation ST3 was chosen to be the most suitable base for the alum, since it contained the least amount of theobroma oil (or more shea butter) and may be the cheapest to produce. The demand for shea butter would boost the economic fortunes of the shea butter industry in Ghana.

### 3.7. Rectoanal Coefficient (RAC) and Histological Findings

Application of croton oil to the anal region has been found to induce inflammation and tissue damage that results in hemorrhoid formation in rats [41]. Hemorrhoid formation is associated with destruction of the mucus epithelium, necrosis of the mucus layer, infiltration of inflammatory cells, and vasodilatation [41, 42].

**Figure 9:**
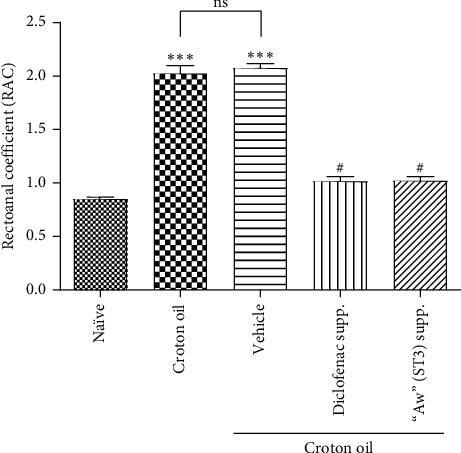
Effect of various treatments on the rectoanal coefficient in croton oil-induced hemorrhoids in rats. Data were expressed as mean ± SEM. ns = not significant, ^*∗∗∗*^*P* < 0.001 when compared to naive and ^#^*P* < 0.05 when compared to vehicle using Kruskal–Wallis nonparametric one-way ANOVA followed by Dunn's *post hoc* test (*n* = 5).

In this work, rectoanal coefficient (RAC), also known as “organ to body weight ratio,” was one of the parameters measured to evaluate the severity of hemorrhoids induced in the rats. RAC is a simple means of comparing the extent of inflammation in the rectoanal tissue [28]. As expected, evaluation of rectoanal coefficient, presented as column graphs (Figure 9), revealed that the croton oil (disease) group showed significantly high RAC (2.03 ± 0.07, *P*=0.0004) when compared to the naïve control (0.852 ± 0.02). Also, as anticipated, the vehicle (bland base) did not have an effect on croton oil-induced hemorrhoids, as the vehicle group showed significantly high RAC (2.08 ± 0.04, *P* < 0.0001) when compared to the naïve and similar to that of the croton oil (disease) control. The results further showed that croton oil-induced hemorrhoid was responsive to anti-inflammatory drug treatment, as the anti-inflammatory drug, diclofenac, significantly reduced the RAC to 1.02 ± 0.04 (*P*=0.0171) when compared to the vehicle (2.08 ± 0.04). Interestingly, the formulated product caused a significant reduction in the RAC to 1.02 ± 0.03 (*P*=0.0203), suggesting that it has an ameliorative effect on croton oil-induced hemorrhoids. These results, although preliminary, are very significant, as Azeemuddin and colleagues [28] have used this same animal model to show the efficacy of the herbal formulations in hemorrhoids.

**Figure 10:**
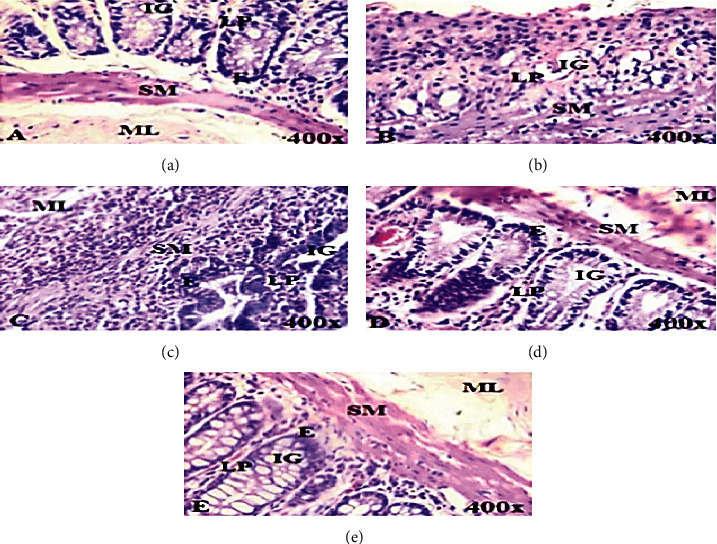
Photomicrographs of the naïve control (a) and croton oil-induced inflammation in the rectoanal tissues in rats (b–e). (a) Histology of the rectoanal tissue of the naïve control group (G1). (b) Histology of the disease control group (G2). (c) Histology of the vehicle (bland base treatment) treatment group (G3). (d) Histology of the diclofenac treatment group (G4) (15 mg/kg bodyweight diclofenac suppository, delivered i.r.). (e) Alum “Aw” suppository treatment group (100 mg/kg bodyweight delivered intrarectally (i.r.)). IG: intestinal gland; LP: lamina propria; E: epithelium; SM: submucosa; ML: muscularis mucosa.

The results obtained from the RAC analysis is further corroborated by the histopathology investigation presented as photomicrographs (Figure 10). Not surprisingly, both the disease control (Figure 10(b)) and vehicle control (Figure 10(c)) showed a very extensive loss of rectoanal integrity, as evidenced by denaturation of the muscularis mucosa (ML), submucosa (SM), and lamina propria (LP) as compared to the naïve (Figure 10(a)). On the other hand, this damage was less severe in the diclofenac-treated (Figure 10(d)) and alum-treated groups (Figure 10(e)), confirming that this model is responsive to anti-inflammatory drug treatment and that alum (Aw) treatment was protective against croton oil-induced hemorrhoids.

Diclofenac, the standard anti-inflammatory drug used in this analysis, is a nonsteroidal anti-inflammatory drug that inhibits cyclooxygenase activity to inhibit inflammation. The current work did not investigate the mechanism of action of alum (Aw) in croton oil-induced hemorrhoids. However, the anti-inflammatory effects of alum are already known. Alum is known to inhibit inflammation via a number of mechanisms, some of which are implicated in croton oil-induced hemorrhoids. The alum effect includes the inhibition of immune cell functions such as the reduction in lymphocyte infiltration, inhibition of goblet cell proliferation, and decrease in dilatation and congestion of blood vessels [26]. These effects may therefore account for the antihemorrhoid effects of the locally produced alum suppositories.

## 4. Conclusions

The alum produced from the bauxite waste from Awaso in Ghana conformed to the British Pharmacopoeia specifications and qualifies as an active pharmaceutical ingredient. There was no interaction between the alum and the bases used as confirmed by the attenuated total reflection Fourier transform infrared (ATR-FTIR) spectroscopy. The combination of shea butter and theobroma oil (3 : 1) as a base ensured the immediate release of the alum from the suppository and passed the pharmacopoeia tests conducted. The produced alum suppositories had antihemorrhoid properties comparable to diclofenac sodium as evidenced by the rectoanal coefficient and the histopathological evaluations. Suppositories containing beeswax showed controlled or slow release properties of the formulations and hence could be used as controlled release suppositories. The alum suppository was successfully formulated and had the appropriate therapeutic outcome.

## Figures and Tables

**Table 1:** Quantities of alum, shea butter, theobroma oil, and beeswax used for the formulation of suppositories.

Formulations	Quantities (g)			
	Alum (Aw)	Shea butter	Theobroma oil	Beeswax
BS1	20	66.22		3.49 (5%)
BS2	20	62.72		6.97 (10%)
BS3	20	58.91		10.40 (15%)
BS4	20	55.28		13.82 (20%)
BS5	20	51.83		17.83 (25%)
BT	20		66.05	3.48 (5%)
TH	20		68.11	
ST1	20	33.75 (1 part)	33.75 (1 part)	
ST2	20	46.03 (2 parts)	23.01 (1 part)	
ST3	20	51.70 (3 parts)	17.24 (1 part)	

BS: beeswax with shea butter; BT: beeswax with theobroma oil; TH: theobroma oil; ST: shea butter with theobroma oil. Stated numbers are based on the preparation of 40 suppositories (*n* = 40).

**Table 2:** Physical characteristics of the alum.

Characteristics	Results: alum (Aw)
Taste	Styptic
Colour	White
Odour	Odourless
Surface appearance	Smooth and glassy
pH	3.29 ± 0.01
Solubility	13.67 ± 0.01 g/100 ml (25°C)
	30.80 ± 0.01 g/100 ml (50°C)
Aluminium (Al^3+^)	Present
Potassium (K^+^)	Present
Sulphate ions (SO_4_^2−^)	Present

The pH and solubility values are presented by mean ± standard deviation (*n* = 3).

**Table 3:** Displacement values of alum (Aw) in the base.

Suppository base	Displacement value
BS1	1.73 ± 0.014
BS2	1.72 ± 0.007
BS3	1.70 ± 0.000
BS4	1.69 ± 0.021
BS5	1.64 ± 0.007
BT	1.76 ± 0.042
TH	1.73 ± 0.028
ST1	1.73 ± 0.014
ST2	1.70 ± 0.007
ST3	1.72 ± 0.000

BS: beeswax with shea butter; BT: beeswax with theobroma oil; TH: theobroma oil; ST: shea butter with theobroma oil. Values are mean ± standard deviation (*n* = 3).

**Table 4:** Quality control evaluation of various suppository formulations.

Formulation	Weight variation (*n* = 20)	Disintegration time (min) (*n* = 6) (mean ± SD)	Content of alum in suppository ± SD (%) (*n* = 10)
BS1	Pass	6.21 ± 0.09	97.97 ± 0.986
BS2	Pass	8.28 ± 0.12	96.46 ± 1.666
BS3	Pass	9.58 ± 0.20	97.41 ± 1.097
BS4	Pass	10.36 ± 0.10	95.04 ± 3.161
BS5	Pass	14.69 ± 0.33	94.41 ± 3.359
BT	Pass	5.26 ± 0.10	99.31 ± 1.446
TH	Pass	3.11 ± 0.14	98.36 ± 3.088
ST1	Pass	2.10 ± 0.07	94.88 ± 3.112
ST2	Pass	2.56 ± 0.17	99.74 ± 2.803
ST3	Pass	3.19 ± 0.08	96.31 ± 2.157

BS: beeswax with shea butter; BT: beeswax with theobroma oil; TH: theobroma oil; ST: shea butter with theobroma oil. SD = standard deviation.

## Data Availability

The data used to support the findings of this study are included in the article and available from the corresponding author upon request.
